# The results of a study on the prevalence of HIV, HCV and HBV genotypes in some regions of Ukraine

**DOI:** 10.1186/1742-4690-9-S1-P55

**Published:** 2012-05-25

**Authors:** Yurchenko, Tetiana Stepchenkova, Iryna Karnets, Kenneth Ashworth, Tetiana Cheusova

**Affiliations:** 1 Kiev Regional Aids Center, Kiev, Ukraine

## Background

Ukraine occupies a leading position in Europe on HIV, HCV and HBV infections. 197083 cases of HIV infection and 2000000 cases of viral hepatitis are officially reported among Ukrainians.

Specialists of the Kiev regional AIDS center and other regional AIDS centers of Ukraine with the assistance of the "EW BIOPHARMA" company (by order of "Aldima SA") are conducting a study on genotyping HIV, HCV and HBV.

## Methods and test-systems

- ELISA testing (Genscreen ULTRA HIV Ag-Ab; Monolisa HCV Ag-Ab ULTRA; Monolisa HBs Ag Ultra)

- PCR testing (Versant HIV-1 RNA 3,0(bDNA); RMS CAP CTM HCV PCR; Abbott Real Time HCV; AmpliprepCOBAS TaqMan HCV Test; AmpliSense HCV Monitor FRT; RMS CAP CTM HBV PCR)

- Genotyping (Trugene HIV-1 Genotype; Versant HCV v2,0 (Lipa); AmpliSense HCV- genotype FL;Quest HBV_Genotype)

Duration of a study - August 2011 - present time.

All patients gave their voluntary consent to involvement and publication of the results. The study includes patients with HIV, or HCV, or HBV. Co-infected patients, which was confirmed by ELISA testing, were excluded.

200 samples have been tested during 4 mounths of 2011. Among them:

- HIV-58

- HCV-121

- HBV-21

In the genotyping of 58 samples of HIV patients were revealed:

- M,A -21 (36,2%)

- M,B -8 (13,8%)

- M-1 (1,7%

- no genotype - 28(48,3%) (undetectable viral load)

In the genotyping of 121 samples of HCV patients were revealed:

- 1a-2(1,6%)

- 1b-51(42,1%)

- 2a-1(0,8%)

- 2ac-1(0,8%)

- 3a-35(28,8%)

- 4-1(0,8%)

- no genotype - 30(25,1%) (undetectable viral load)

In the genotyping of 21 samples of HBV patients were revealed:

- A-3(14,2%)

- C-1(4,7%)

- D-11(52,4%)

- no genotype - 6(28,7%) (undetectable level of viral load)

## Conclusion

Among the samples of plasma of HIV patients, the most common genotype is -M,A (36,2%)

Among the samples of plasma of patients with HCV, the most common genotype is 1b -(42,1%)

Among the samples of plasma of patients with HBV, the most common genotype is D-(52,4%)

